# 1340. Towards better understanding of the epidemiology of SARS-CoV-2 PCR conversion in hospitalized patients: a case-control study

**DOI:** 10.1093/ofid/ofad500.1177

**Published:** 2023-11-27

**Authors:** Farrin A Manian, Chaitanya A Karlapalem

**Affiliations:** Mercy-St. Louis, St. Louis, Missouri; Mercy-St. Louis, St. Louis, Missouri

## Abstract

**Background:**

The epidemiology of hospitalized patients who "convert" from a negative nasopharyngeal SARS-CoV-2 by PCR to a positive test during the same hospitalization has not been adequately studied.

**Methods:**

This was a retrospective case-control study at 2 large suburban community hospitals. Eligible adult inpatients 18 years or older who underwent 2 or more nasopharyngeal SARS-CoV-2 PCR testing during the same hospital stay from January 1, 2020-March 31, 2021 were included. Controls were randomly selected from a data base of patients with negative repeat test(s) during the same hospitalization at respective hospitals (case-control ratio 1:1)

**Results:**

Of 40 SARS-CoV-2 PCR converters (18 cases from hospital A, 22 cases from hospital B), 23 (57.5%) were 65 years or older, 21 (52%) were female, and 36 (90%) were Caucasian; mean BMI was 28.4. Thirteen (32.5%) patients had 1 or more common Covid-19 symptoms (eg, cough, fever, shortness of breath, weakness, GI symptoms, loss of appetite, confusion) on initial testing (98% within 24 hours of admission). Mean interval between paired tests was 8.4 days; 52.5% were repeated on hospital day 7 or later. Compared to non-converters, converters were less likely to be 65 y or older (58% vs 80%), more likely to be retested due to new Covid-19 symptoms (35% vs 7.5%), had longer hospital stay (17.8 days vs 9.6 days) and had higher in-hospital mortality (32.5% vs zero) (P< 0.001 for all). There was no significant difference between converter and non-converters in the prevalence of Covid-19 symptoms on initial testing, sex, race, BMI, time spent in the ED or other hospital wards, prevalence of selected high-risk Covid-19 comorbidities (eg, hypertension, diabetes, immunocompromised state, COPD, chronic kidney disease or cancer) or interval between paired tests.

**Conclusion:**

Hospitalized patients who convert from a negative to a positive nasopharyngeal SARS-CoV-2 PCR test during the same hospitalization are more likely to be less than 65 y old, be retested because of new-onset Covid-19 symptoms often a week or more days into hospitalization and have significantly longer hospital stay and higher inpatient mortality compared to those of non-converters. More studies are needed to determine factors associated with SARS-Cov-2 PCR conversion in this vulnerable patient population.

**Disclosures:**

**All Authors**: No reported disclosures

